# Exploring the association between atmospheric pollutants and preterm birth risk in a river valley city

**DOI:** 10.3389/fpubh.2024.1415028

**Published:** 2024-07-25

**Authors:** Jiajia Gu, Jimin Li, Lang Liu, Meiying Cao, Xi Tian, Zeqi Wang, Jinwei He

**Affiliations:** Medical School of Yan’an University, Yan’an, China

**Keywords:** preterm birth, air pollution, environmental exposure, risk assessment, public health

## Abstract

**Objective:**

To investigate the association between exposure to atmospheric pollutants and preterm birth in a river valley-type city and its critical exposure windows.

**Methods:**

A retrospective cohort study was used to collect data from the medical records of preterm and full-term deliveries in two hospitals in urban areas of a typical river valley-type city from January 2018 to December 2019. A total of 7,288 cases were included in the study with general information such as pregnancy times, the number of cesarean sections, occupation, season of conception and regularity of the menstrual cycle. And confounding factors affecting preterm birth were inferred using the chi-square test. The effects of exposure to each pollutant, including particulate matter 2.5 (PM_2.5_), particulate matter 10 (PM_10_), nitrogen dioxide (NO_2_), sulfur dioxide (SO_2_), carbon monoxide (CO) and ozone (O_3_), during pregnancy on preterm birth and the main exposure windows were explored by establishing a logistic regression model with pollutants introduced as continuous variables.

**Results:**

Maternal age, pregnancy times, number of births, number of cesarean sections, season of conception, complications diseases, comorbidities diseases, hypertension disorder of pregnancy and neonatal low birth weight of the newborn were significantly different between preterm and term pregnant women. Logistic regression analysis after adjusting for the above confounders showed that the risk of preterm birth increases by 0.9, 0.6, 2.4% in T_2_ and by 1.0, 0.9, 2.5% in T_3_ for each 10 μg/m^3^ increase in PM_2.5_, PM_10,_ NO_2_ concentrations, respectively. The risk of preterm birth increases by 4.3% in T_2_ for each 10 μg/m^3^ increase in SO_2_ concentrations. The risk of preterm birth increases by 123.5% in T_2_ and increases by 188.5% in T_3_ for each 10 mg/m^3^ increase in CO concentrations.

**Conclusion:**

Maternal exposure to PM_2.5_, PM_10,_ NO_2_, CO was associated with increased risk on preterm birth in mid-pregnancy (T_2_) and late pregnancy (T_3_), SO_2_ exposure was associated with increased risk on preterm birth in mid-pregnancy (T_2_).

## 1 Introduction

There are approximately 13.4 million preterm births globally in 2020, accounting for more than one-tenth of all newborns (1). Although the number of preterm births has declined compared to 2010, there has been no measurable change in the global preterm birth rate during this decade (1). In China, the status of preterm birth is not encouraging. Research data show that China’s preterm birth rate is 12.0% in 2014, the second highest in the world (2). With the opening of China’s two-child policy in 2016, the preterm birth rate has shown a trend of gradual increase (3), and the incidence is not balanced between regions (4).

Rising rates of preterm birth are accompanied by an increase in the number of children under the age of five who die from preterm birth complications, with statistics indicating that approximately 900,000 children worldwide have died from preterm birth complications in 2019 (5). The infants who survive from preterm birth events also face great risks, such as lifelong disabilities, and these surviving preterm infants are prone to comorbidities such as cerebral palsy, progressive developmental lag, chronic lung disease or neurological sequelae (6, 7), which can impose a heavy burden on both families and society in terms of mental and economic aspects. Preterm labor is considered to be triggered by multiple mechanisms, including infection or inflammation, uteroplacental ischemia or hemorrhage, uterine overstretching, stress, oxidative stress, and other immune-mediated processes (8, 9). Besides, there is evidence that preterm birth is the result of the interaction of multiple risk factors (10), and in addition to well-known risk factors such as maternal demographics (11, 12), psychological characteristics (13), pregnancy comorbidities (14), and genetic characteristics (15), epidemiological studies have suggested that preterm birth is associated with atmospheric pollutants (16–18).

In China, with the rapid economic development of industrialization and urbanization in the past decades, environmental problems have become increasingly serious (19). These are dominated by increasing atmospheric pollution and particulate matter in the environment, with PM_2.5_, PM_10_, SO_2_, O_3_, NO_2_ and CO being the main air pollutants. A study on air pollutants conducted in 2015 found that the rate of air pollution and persistent air pollution in northern China is much higher than that in the south, especially in cities in the Bohai Rim and Xinjiang Province (20). It can be seen that there is spatial heterogeneity in air pollutant levels in different cities, especially in the northern cities of China. Therefore, a typical river valley city located in Northwest China was selected for this study, which develops on the axis of the Weihe Plain and is dominated by mountains and hills, with a slightly more complex geological structure than other surrounding cities. The city has a long heating period due to cold winters, which increases the amount of coal and carbon consumed, and the pollutants released from coal combustion are not easily dispersed due to the unique geographic characteristics of the city. It is also due to the frequent occurrence of unfavorable weather such as fog and inversions, which further contribute to the increase in pollutant concentrations.

This study investigates the association between exposure to pollutants and the occurrence of preterm birth in river basin cities and the main exposure windows, with a view to inform potential risk factors of preterm births.

## 2 Materials and methods

### 2.1 Study participants

We collect information on all pregnant women with preterm and full-term births from January 2018 to December 2019 from two hospitals in Baoji city. This includes general maternal information (name, age, date of admission, occupation, gestational address), current pregnancy (pregnancy times, number of births, number of cesarean sections, last menstrual period, season, regularity of menstrual cycle, mode of delivery in this case), pregnancy outcome, neonatal information [neonatal date of birth, gestational age (gestational age was usually calculated from the first day of the mother’s last menstrual period), weight (g), number of births], complications diseases, hypertensive disorder of pregnancy, comorbidity diseases and passive smoking.

Pregnancy comorbidities are a condition in which a pregnant woman develops other diseases in addition to the symptoms associated with pregnancy, i.e., a state in which a pregnant woman is comorbid with one or more diseases. Including combined cardiovascular disease, combined hematological disease, combined respiratory disease, combined gastrointestinal disease, combined urological disease, combined endocrine disease, combined infectious disease and combined neoplasm.

Pregnancy complications refer to a variety of conditions that occur during pregnancy that may have some impact on the health of the mother and the births. These include placenta previa, placental abruption, premature rupture of membranes, low amniotic fluid, fetal distress and so on.

Inclusion criteria: ① local residence for more than 1 year and detailed address; ② no assisted conception (exclusion of fertility achieved through unnatural conception and with the help of medical technology); ③ age greater than 18 years old; ④ no acute or chronic diseases; ⑤ the births was born as a single live birth; ⑥ no communication barriers (communication barriers, including hearing or visual impairments, neurological disorders or psychological disorders, etc.). Exclusion criteria: ① not residing in Baoji city during pregnancy or residing locally for less than 1 year; ② assisted conception (e.g., *in vitro* fertilization (IVF)); ③ unmarried women; ④ ectopic pregnancy; ⑤ births born as twin or multiple births.

In the case information collected in this retrospective cohort study, a total of 7,288 cases were included in the study, of which 372 cases were preterm pregnant women and 6,916 cases were full-term pregnant women. Preterm births were selected from those delivered at 28 weeks to less than 37 weeks of gestation from the first day of the last menstrual period, and term births were selected from those delivered at 37 weeks of gestation.

The questionnaire used to collect information on maternity was designed with a clear research objective combined with existing research findings and expertise. A small-scale pre-test was conducted before its official use, and the questionnaire was further revised and improved based on the test results. Validity analyses were also conducted to assess the reasonableness of the questionnaire design as well as to verify the reliability of the questionnaire through reliability analyses. Finally, in order to ensure the high quality of the information collected, all investigators were required to receive professional training before entering the above hospitals, obtaining the case records of all participants, and completing the questionnaire.

### 2.2 Methods

#### 2.2.1 Air pollutant exposure assessment

**Figure 1:**
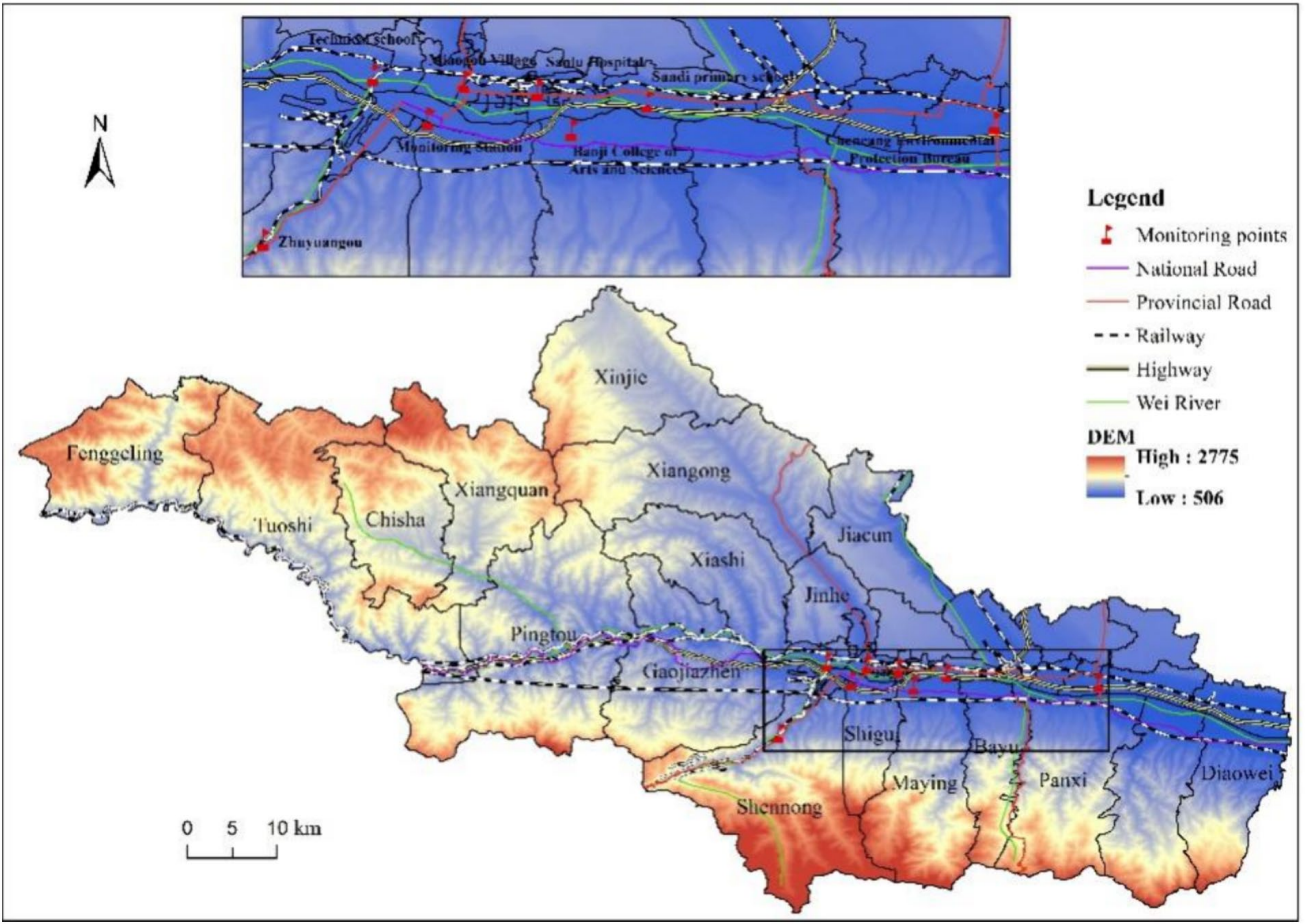
Distribution of air quality monitoring stations and topographic map of Baoji city.

A total of 7 air monitoring stations have been set up in Baoji city, the distribution of which can be seen at Figure 1. The actual straight-line distance between the pregnant women eventually included in the study and each of the above air monitoring stations was calculated based on the latitude and longitude of their main residential address during pregnancy, and then the nearest monitoring station to each of the pregnant women’s residential address was selected. The air pollutant concentrations monitored at that station were used as the exposure of air pollutants for that pregnant woman during her pregnancy period. The air pollutant data was obtained from the National Urban Air Quality Real-Time Distribution System (https://air.cnemc.cn:18007/) of the China Environmental Monitoring General Station of the Ministry of Environmental Protection (MEP), and included the concentrations of six pollutants, namely, PM_2.5_, PM_10_, O_3_, SO_2_, CO and NO_2_. The PM_2.5_, PM_10_, SO_2_, CO and NO_2_ are 24 h moving averages, and O_3_ is the maximum 8 h moving average. To determine the pollutant exposure window, we divided the course of pregnancy into three stages: 0–12 weeks of gestation was defined as early pregnancy (T_1_), 13–27 weeks as mid-pregnancy (T_2_), and 28 weeks to the end of pregnancy as late pregnancy (T_3_) (21, 22). According to the time period corresponding to the different exposure windows of each pregnant women, the daily moving average of each pollutant in that time period was found separately, and after averaging, the air pollutant exposure level of the pregnant women in a certain exposure window was modelled accordingly, so as to determine the susceptibility windows for various air pollutants during pregnancy.

#### 2.2.2 Statistical methods

Data was collated using Excel and analyzed using SPSS 20.0. Normality test was performed for pollutants and the levels of exposure to pollutants in different exposure windows were described using mean, standard deviation (SD), median, and interquartile range (IQR). General maternal data was compared using one-way chi-square test to determine whether there is a difference between pregnant women with preterm versus term births. If they do, they can be considered and evaluated as potential confounders and were introduced as covariates in the subsequent logistic regression model. After the test, confounding factors are maternal age, pregnancy times, number of births, number of cesarean sections, season of conception, complications disease, comorbidity diseases, hypertensive disorder complicating pregnancy and low birth weight. For missing data, it was treated as discrete missing values 999 in SPSS software. The relationship between each pollutant and preterm birth and the main exposure windows were explored by logistic regression modelling, adjusting for confounding factors after introducing the pollutant as a continuous variable (α = 0.05).

## 3 Results

### 3.1 General description of air pollution in Baoji city 2017–2019

**Figure 2:**
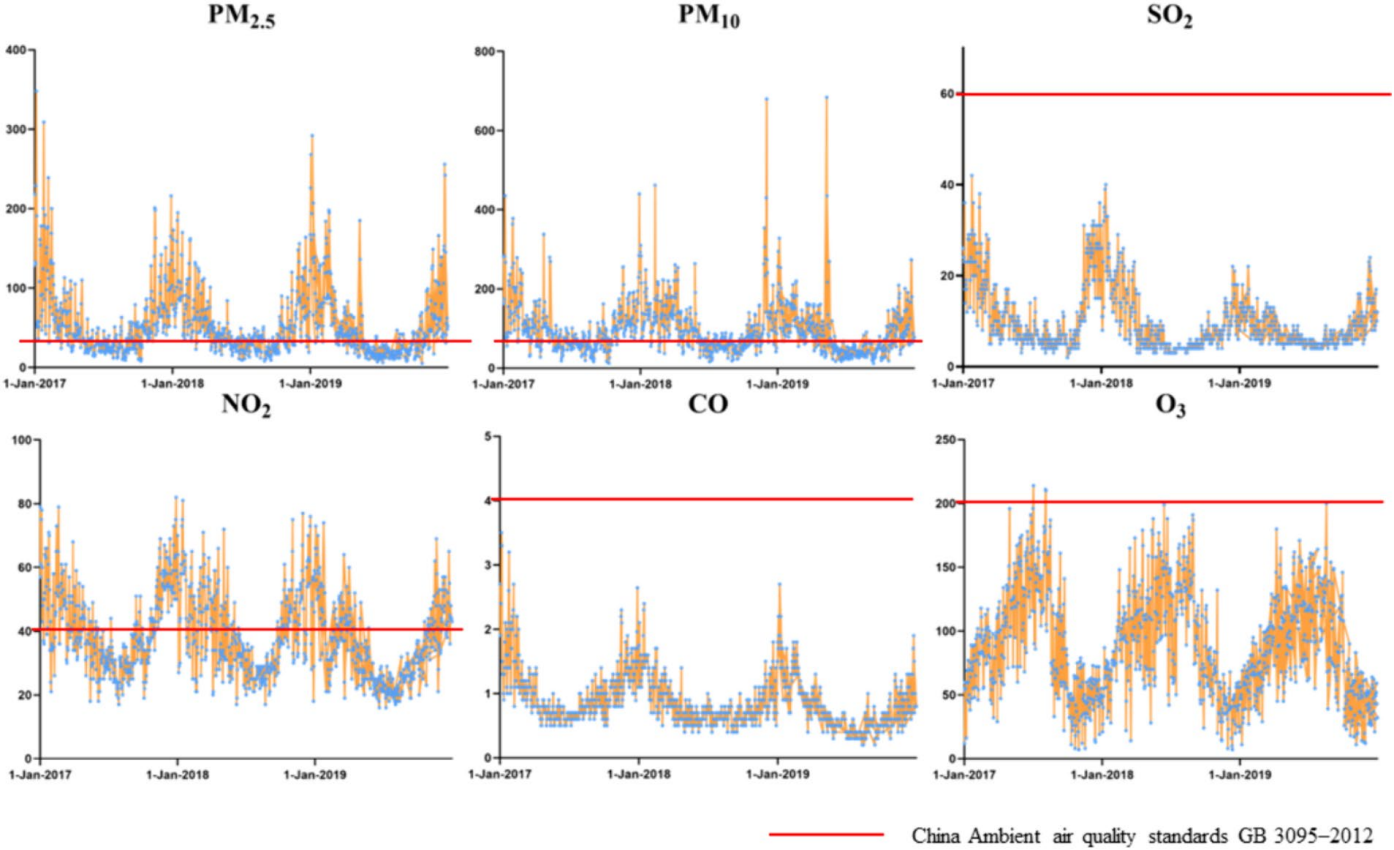
Daily variation of air pollutant concentrations in Baoji city, 2017–2019.

The air pollutant exposure window for some of the pregnant women who gave birth in 2018 was in 2017, so 2017 was included when describing the air pollution profile. And we compared the concentration of each pollutant with the national secondary standard (Level II), respectively. The national secondary standards of PM_2.5_, PM_10_, SO_2_, NO_2_, CO and O_3_ are respectively 35 μg/m^3^, 70μg/m^3^, 60μg/m^3^, 40μg/m^3^, 4mg/m^3^, 200μg/m^3^ (Table 1). During the period of 2017–2019, PM_2.5_ and PM_10_ concentrations were significantly higher than the national secondary standards most of the time; SO_2_ and CO concentrations were always lower than the national secondary standards; NO_2_ concentrations showed an unstable situation of being sometimes higher and sometimes lower than the national secondary standards; and O_3_ concentrations, although they were almost always lower than the national secondary standards, showed a different pattern from the other pollutants: with peaks in the summer and drops in the winter (Figure 2).

**Table 1:** Description of exposure levels in preterm and term women across exposure windows in 2017–2019.

Pollutants	Preterm birth				Full term birth				China ambient air quality standards GB 3095–2012
	Mean	SD	Median	IQR	Mean	SD	Median	IQR	
Trimester 1									Level II
PM_2.5_ (μg/m^3^)	54.67	24.82	47.29	46.88	53.79	24.18	46.82	45.14	35
PM_10_ (μg/m^3^)	100.71	31.16	103.37	56.59	99.48	31.37	100.89	61.32	70
SO_2_ (μg/m^3^)	8.81	3.99	7.56	4.31	8.83	4.06	7.85	4.25	60
NO_2_ (μg/m^3^)	37.13	8.06	35.84	14.12	37.29	8.14	35.98	14.19	40
CO (mg/m^3^)	0.88	0.23	0.8	0.42	0.87	0.23	0.79	0.41	4
O_3_ (μg/m^3^)	86.03	32.85	90.65	56.51	85.22	35.15	89.64	60.57	200
Trimester 2									#
PM_2.5_ (μg/m^3^)	50.88	25.48	41.88	45.02	54.51	25.36	49.19	47.64	#
PM_10_ (μg/m^3^)	94.64	34.06	91.49	62.68	99.50	32.96	102.93	61.39	#
SO_2_ (μg/m^3^)	8.52	4.37	6.97	4.85	8.80	4.24	7.59	4.86	#
NO_2_ (μg/m^3^)	35.73	9.46	34.52	15.77	36.96	9.06	36.81	14.55	#
CO (mg/m^3^)	0.83	0.28	0.71	0.43	0.86	0.26	0.84	0.46	#
O_3_ (μg/m^3^)	83.80	33.46	96.1	57.69	80.44	32.19	85.39	57.24	#
Trimester 3									#
PM_2.5_ (μg/m^3^)	52.17	28.51	42.82	51.69	53.63	27.45	43.64	51.32	#
PM_10_ (μg/m^3^)	95.42	39.42	93.73	74.95	97.62	36.45	98.64	69.04	#
SO_2_ (μg/m^3^)	8.71	4.75	6.93	4.72	8.77	4.49	7.35	4.76	#
NO_2_ (μg/m^3^)	35.97	10.36	35.82	17.45	36.25	9.65	35.66	16.38	#
CO (mg/m^3^)	0.80	0.30	0.69	0.44	0.82	0.29	0.69	0.46	#
O_3_ (μg/m^3^)	82.79	31.85	88.48	53.67	83.23	30.87	90.25	52.72	#

0–12 weeks of gestation was defined as early pregnancy (T_1_), 13–27 weeks as mid-pregnancy (T_2_), and 28 weeks to the end of pregnancy as late pregnancy (T_3_). #: No data or information at this location. Level II is a standard, which means acceptable air quality, but some pollutants may have a weak impact on the health of a very small number of unusually sensitive people.

### 3.2 Comparative analysis of general maternal information

Analysis of the general data of pregnant women revealed that: pregnant women older than 30 years are more likely to have preterm births; pregnant women with more than one pregnancy times, births and cesarean sections are prone to have preterm births; compared with full-term pregnant women, season of conception of preterm pregnant women is concentrated in winter (29.3%) and spring (27.7%); the proportion of preterm pregnant women suffering from pregnancy complications (93.3%), hypertensive disorders of pregnancy (18.8%), and pregnancy comorbidities (77.7%) is higher than that of full-term pregnant women. At the same time, preterm births are more likely to result in low birth weight (46.2%).

Maternal age, pregnancy times, number of births, number of cesarean sections, season of conception, complications diseases, comorbidities diseases, hypertension disorder of pregnancy, and neonatal low birth weight of the newborn were significantly (*P* < 0.05) different between preterm and term pregnant women. Maternal occupation, regularity of the menstrual cycle and passive smoking did not differ significantly between preterm and term pregnant women. And we did not model newborn sex and other variables, such as mother’s education and maternal smoking during pregnancy, because of the difficulty to obtain the data in retrospective cohort study and large number of missing values (Table 2).

**Table 2:** Comparative analysis of the general data of preterm and term pregnant women.

Covariates		Preterm birth		Full term birth		*p*
		(*n* = 372)		(*n* = 6,916)		
		*N*	*P* (%)	*N*	*P* (%)	
Mean maternal age		30.07		29.29		*#*
Maternal age range		20–43		18–46		*#*
Maternal age (years)	18–19	0	0.0	22	0.3	0.007
	20–24	40	10.8	795	11.5	
	25–29	144	38.7	3,089	44.7	
	30–34	123	33.1	2,201	31.8	
	35	65	17.5	809	11.7	
Pregnancy times	1	102	27.4	2,436	35.2	0.002
	>1	270	72.6	4,478	64.7	
Number of births	≤1	174	46.8	3,789	54.8	0.002
	>1	198	53.2	3,118	45.1	
Number of cesarean sections	≤1	280	75.3	5,587	80.8	0.008
	>1	92	24.7	1,326	19.2	
Occupation	National civil servants	4	1.1	117	1.7	0.373
	Professional and technical staff	58	15.6	1,162	16.8	
	Business and services	84	22.6	1,241	17.9	
	Agriculture	63	16.9	1,338	19.3	
	Production and transport	8	2.2	164	2.4	
	Other special occupations	66	17.7	1,163	16.8	
	Unemployed	73	19.6	1,312	19	
Season of conception	Spring	103	27.7	1,712	24.8	0.034
	Summer	85	22.8	1,774	25.7	
	Fall	75	20.2	1,726	25	
	Winter	109	29.3	1,704	24.6	
Regularity of the menstrual cycle	Regularity	366	98.4	6,791	98.2	0.601
	Irregularity	4	1.1	97	1.4	
Pregnancy complications	Yes	347	93.3	6,077	87.9	0.002
	No	25	6.7	839	12.1	
Hypertensive disorder of pregnancy	Yes	70	18.8	353	5.1	0.000
	No	261	70.2	5,751	83.2	
Pregnancy comorbidities	Yes	289	77.7	4,804	69.5	0.001
	No	83	22.3	2,112	30.5	
Low birth weight	≥2,500 g	197	53	6,808	98.4	0.000
	<2,500 g	172	46.2	84	1.2	
Passive smoking	Yes	10	2.7	160	2.3	0.769
	No	274	73.7	4,833	69.9	

*P* (%) means percentage of the number of people with each categorical factor in the preterm and term birth groups, respectively. *p*-value comes from the chi-square test, at the significance level of *α* = 0.05, the smaller the *p*-value, the more reason there is to believe that a factor differs between the two groups of pregnant women. Pregnancy times include the current pregnancy. Pregnancy comorbidities are a condition in which a pregnant woman develops other diseases in addition to the symptoms associated with pregnancy. Pregnancy complications refer to a variety of conditions that occur during pregnancy that may have some impact on the health of the mother and the births. #: No data or information at this location.

### 3.3 Comparison of pollutant exposure concentrations in preterm pregnant women versus term pregnant women

In early pregnancy, mid-pregnancy and late pregnancy, the mean levels of exposure concentrations of PM_2.5_ and PM_10_ for preterm and full-term pregnant women were higher than the national secondary standard (Level II), while the concentrations of other pollutants were lower than the national secondary standard.

In early pregnancy, the mean levels of PM_2.5_, PM_10_, CO and O_3_ exposure of preterm pregnant women were 54.67 μg/m^3^, 100.71 μg/m^3^, 0.88 mg/m^3^ and 86.03 μg/m^3^, respectively, which were slightly higher than those of term pregnant women. The mean levels of SO_2_ and NO_2_ exposure were higher in term than in preterm women. In mid-pregnancy, the mean levels of PM_2.5_, PM_10_, SO_2_, NO_2_ and CO exposure for preterm pregnant women were lower than those for term pregnant women, and only the mean level of O_3_ exposure was higher than that for term pregnant women, at 83.80 μg/m^3^. In the late pregnancy, the mean levels of PM_2.5_, PM_10_, SO_2_, NO_2_, CO and O_3_ exposure of preterm women were all lower than those of term pregnant women. Differences in pollutant exposure levels in the three exposure windows between the two groups may be due to the fact that full-term pregnant women are generally exposed for a longer period of time in late pregnancy than preterm pregnant women, which results in higher exposure levels of each pollutant for full-term pregnant women than for preterm pregnant women in late pregnancy (Table 1).

### 3.4 Logistic regression analysis of preterm birth

Before adjustment, only PM_2.5_, PM_10,_ NO_2_, CO exposure was associated with increased risk on preterm birth in mid-pregnancy, and exposure to the other pollutants had no association with preterm birth in each exposure window. In multivariate analyses of single-pollutant models, exposure to PM_2.5_, PM_10,_ NO_2_, CO was associated with increased risk on preterm birth in mid-pregnancy (T_2_) and late pregnancy (T_3_), SO_2_ exposure was associated with increased risk on preterm birth in mid-pregnancy (T_2_). The risk of preterm birth increased by 0.9, 0.6, 2.4% in T_2_ and by 1.0, 0.9, 2.5% in T_3_ for each 10 μg/m^3^ increase in PM_2.5_, PM_10_, NO_2_ concentrations, respectively. The risk of preterm birth increased by 4.3% in T_2_ for each 10 μg/m^3^ increase in SO_2_. The risk of preterm birth increased by 123.5% in T_2_ and by 188.5% in T_3_ for each 10 mg/m^3^ increase in CO concentrations. Other pollutants were not associated with increased risk on preterm birth in 3 exposure windows (Table 3).

**Table 3:** Associations between pollutants and preterm birth.

Pollutants	Unadjusted			Adjusted		
	OR	95% CI	*p*	OR	95% CI	*p*
*Trimester 1*						
PM_2.5_ (μg/m^3^)	0.999	0.994–1.003	0.495	0.991	0.983–0.999	0.034
PM_10_ (μg/m^3^)	0.999	0.995–1.002	0.463	0.993	0.987–0.999	0.023
SO_2_ (μg/m^3^)	1.002	0.976–1.028	0.904	0.984	0.947–1.023	0.426
NO_2_ (μg/m^3^)	1.003	0.990–1.016	0.701	0.994	0.972–1.015	0.553
CO (mg/m^3^)	0.911	0.582–1.425	0.683	0.730	0.335–1.588	0.427
O_3_ (μg/m^3^)	0.999	0.996–1.002	0.667	0.999	0.994–1.004	0.674
*Trimester 2*						
PM_2.5_ (μg/m^3^)	1.006	1.002–1.010	0.007	1.009	1.003–1.014	0.001
PM_10_ (μg/m^3^)	1.004	1.001–1.008	0.006	1.006	1.002–1.010	0.002
SO_2_ (μg/m^3^)	1.016	0.991–1.043	0.210	1.043	1.009–1.077	0.011
NO_2_ (μg/m^3^)	1.015	1.003–1.027	0.011	1.024	1.009–1.038	0.002
CO (mg/m^3^)	1.743	1.167–2.603	0.007	2.235	1.358–3.678	0.002
O_3_ (μg/m^3^)	0.997	0.993–1.000	0.051	0.992	0.987–0.996	0.000
*Trimester 3*						
PM_2.5_ (μg/m^3^)	1.002	0.998–1.006	0.319	1.010	1.002–1.018	0.011
PM_10_ (μg/m^3^)	1.002	0.999–1.005	0.260	1.009	1.003–1.015	0.002
SO_2_ (μg/m^3^)	1.003	0.980–1.027	0.806	1.061	0.999–1.126	0.053
NO_2_ (μg/m^3^)	1.003	0.992–1.014	0.589	1.025	1.001–1.051	0.044
CO (mg/m^3^)	1.324	0.919–1.907	0.131	2.885	1.377–6.042	0.005
O_3_ (μg/m^3^)	1.000	0.997–1.004	0.790	1.001	0.994–1.008	0.770

What increase in pollutant are the results reported for “per 10 μg/m^3^” or “per 10 mg/m^3^.” Adjustment factors: maternal age, pregnancy times, number of births, number of cesarean sections, season of conception, complications disease, comorbidity diseases, hypertensive disorder of pregnancy, low birth weight.

## 4 Discussion

In this retrospective cohort study, we investigated the association between air pollutants and preterm birth in Baoji city in 2018–2019. The study showed that preterm births were conceived more often in spring and winter compared to full-term births. This may be due to the fact that Baoji city has a warm-temperate monsoon climate with cold and dry winters, so coal combustion increases during the collective heating phase and pollutants are released, which results in higher concentrations of air pollutants in spring and winter than in other seasons. There were significant differences between preterm births and full-term births with respect to maternal age, pregnancy times, number of births, number of cesarean sections, season of conception, complications diseases, comorbidities diseases, hypertension disorder of pregnancy and neonatal low birth weight. In multivariate single pollutant models, exposure to PM_2.5_, PM_10,_ NO_2_, CO was associated with increased risk on preterm birth in mid-pregnancy and late pregnancy, and SO_2_ exposure was associated with increased risk on preterm birth in mid-pregnancy. The risk of preterm birth increased by 0.9, 0.6, 2.4% in T_2_ and by 1.0, 0.9, 2.5% in T_3_ for each 10 μg/m^3^ increase in PM_2.5_, PM_10,_ NO_2_ concentrations, respectively. The risk of preterm birth increased by 4.3% in T_2_ for each 10 μg/m^3^ increase in SO_2_. The risk of preterm birth increased by 123.5% in T_2_ and by 188.5% in T_3_ for each 10 mg/m^3^ increase in CO concentrations. There was no association between exposure to O_3_ and preterm birth in any stage of pregnancy.

These results are consistent with findings from other studies. The exposure of PM_2.5_ in T_1_, T_2_, T_3_ and E can increase the risk of preterm birth (23). And the strongest association was observed in the second trimester (24). Exposure to high concentrations of PM_10_ increases the risk of preterm birth (25, 26), the study (25) also suggested that the risk may vary according to the clinical subtypes of preterm birth and the time window of exposure. Significant association was found between NO_2_ exposure and preterm birth (27, 28) and NO_2_ exposure in only the 3rd trimester was positively associated with PTB (29). It is showed that (30) exposure to PM_2.5_, PM_10_, and NO_2_ for 1 week prior to delivery increased the risk of preterm birth. One study (31), also conducted in a river valley type city, showed that PM_10_, O_3_ exposure in late pregnancy, SO_2_ in mid pregnancy, and SO_2_ exposure in late pregnancy were all likely to increase the risk of preterm birth. Although the above studies have shown that exposure to PM_2.5_, PM_10,_ NO_2_, CO and SO_2_ during pregnancy increases the risk of preterm birth, the key exposure windows and associated intensities are not the same, which may be attributed to different pollution levels, specific study periods and study populations, or other factors.

In our study, we did not observe any association for O_3_, however other studies showed that O_3_ was associated with preterm birth. A study (32) showed that exposure to O_3_ during pregnancy increased the risk of preterm birth. Another study in China (33) also illustrated that O_3_ was one of the risk factors for the occurrence of preterm birth, and the susceptibility window was late in pregnancy at T_3_. These inconsistencies may be due to differences in the locations of the studies, differences in the experimental design of the studies, or differences in the methods of exposure assessment and statistical analyses.

Although this study showed that exposures to certain pollutants in later pregnancy is associated with preterm birth, there is no clear understanding of the molecular mechanism of the occurrence of preterm birth induced by air pollutants. It has been suggested that it is fetal growth and development within the placenta that is more sensitive and vulnerable to exposure to air pollutants (34), thereby predisposing to preterm birth or other adverse pregnancy outcomes. Prenatal exposure to air pollution has also been found to be associated with nitrosative stress and epigenetic alterations in the placenta (35). Air pollution particles may be transferred into and across the placental barrier, leading to placental oxidative and nitrosative stress due to the ability of pollution particles to produce reactive oxygen species/reactive nitrogen species (ROS/RNS) in a direct or indirect manner; another important target of the early life effects of air pollution is the induction of epigenetic alterations of the placenta, including DNA methylation, histone and noncoding RNA modifications, and changes in chromatin remodeling. The Developmental Origins of Health and Disease (DOHaD) hypothesis similarly suggests an association between perinatal complications induced by prenatal exposure to air pollutants (preterm birth or fetal growth restriction), and placental epigenomics (36). In contrast, another study found that the onset of preterm birth inversely enhances the toxicity of air pollutants through oxidative stress and placental function (37), which means that air pollution particles affect the anatomical structure and/or physiological function of the developing lungs and related systems through oxidative stress, which also contributes to placental aging leading to preterm birth, and that the occurrence of preterm birth during this critical period may further enhance the ensuing alterations in lung function and physiology.

The strength of this study is that all data was collected from two of the largest hospitals in the city, which are the top hospitals in China’s healthcare system, with a high level of medical technology and quality of service. As a result, large amounts of medical data can be collected and the data tends to be more accurate and reliable, contributing to more accurate conclusions. This study also has some limitations. First, using ambient monitoring data, we could not account for the differences in pollutant concentrations between the daily living and working environments of the study subjects. And assessing pollutant exposure levels of the study subjects based only on their home addresses may lead to some errors and may bias the association; Secondly, although the nearest monitoring site method is able to obtain more accurate data than the global average method used in previous studies, it still has some limitations. For example, in some cases, the quality of data collected may be poor due to errors in monitoring equipment, improper maintenance, etc., affecting the accuracy of the analyzed results. In addition, the large number of missing important confounders, such as child sex, maternal education level, and marital status is an important limitation of this study.

## 5 Conclusion

In conclusion, we observed an association between exposures to PM_2.5_, PM_10_, NO_2_, CO in mid-pregnancy and late pregnancy and increased risk on preterm birth, but little evidence of associations with O_3_.

In light of importance of air quality on maternal health and birth outcomes, measures to improve air quality, its monitoring and health educations for women especially in reiver valley cities need to be public health priorities.

## Data availability statement

The original contributions presented in the study are included in the article/supplementary material, further inquiries can be directed to the corresponding author.

## Ethics statement

The studies involving humans were approved by Medical School of Yan’an University. The studies were conducted in accordance with the local legislation and institutional requirements. The participants provided their written informed consent to participate in this study. Written informed consent was obtained from the individual(s) for the publication of any potentially identifiable images or data included in this article.

## Author contributions

JG: Conceptualization, Data curation, Formal analysis, Investigation, Methodology, Project administration, Writing – original draft, Writing – review & editing. JL: Conceptualization, Formal analysis, Investigation, Methodology, Writing – original draft, Writing – review & editing. LL: Investigation, Methodology, Project administration, Software, Writing – original draft, Writing – review & editing. MC: Investigation, Methodology, Project administration, Software, Writing – original draft, Writing – review & editing. XT: Data curation, Methodology, Writing – original draft, Writing – review & editing. ZW: Data curation, Methodology, Writing – original draft, Writing – review & editing. JH: Funding acquisition, Supervision, Writing – original draft, Writing – review & editing.
